# Two-Photon Excitation Spectra of Various Fluorescent Proteins within a Broad Excitation Range

**DOI:** 10.3390/ijms232113407

**Published:** 2022-11-02

**Authors:** Ruth Leben, Randall L. Lindquist, Anja E. Hauser, Raluca Niesner, Asylkhan Rakhymzhan

**Affiliations:** 1 Biophysical Analytics, Deutsches Rheuma-Forschungszentrum (DRFZ), 10117 Berlin, Germany; 2 Institute of Immunology, Center for Infection Medicine, Freie Universität Berlin, 14163 Berlin, Germany; 3 Immune Dynamics and Intravital Microscopy, Deutsches Rheuma-Forschungszentrum (DRFZ), 10117 Berlin, Germany; 4 Praxen für Nuklearmedizin, 12163 Berlin, Germany; 5 Rheumatology and Clinical Immunology, Charité–Universitätsmedizin Berlin, 10117 Berlin, Germany; 6 Dynamic and Functional In Vivo Imaging, Freie Universität Berlin, 14163 Berlin, Germany

**Keywords:** two-photon excitation spectrum, two-photon fluorescence laser-scanning microscopy, cells expressing fluorescent proteins, HEK-293T cells and murine splenocytes

## Abstract

Two-photon excitation fluorescence laser-scanning microscopy is the preferred method for studying dynamic processes in living organ models or even in living organisms. Thanks to near-infrared and infrared excitation, it is possible to penetrate deep into the tissue, reaching areas of interest relevant to life sciences and biomedicine. In those imaging experiments, two-photon excitation spectra are needed to select the optimal laser wavelength to excite as many fluorophores as possible simultaneously in the sample under consideration. The more fluorophores that can be excited, and the more cell populations that can be studied, the better access to their arrangement and interaction can be reached in complex systems such as immunological organs. However, for many fluorophores, the two-photon excitation properties are poorly predicted from the single-photon spectra and are not yet available, in the literature or databases. Here, we present the broad excitation range (760 nm to 1300 nm) of photon-flux-normalized two-photon spectra of several fluorescent proteins in their cellular environment. This includes the following fluorescent proteins spanning from the cyan to the infrared part of the spectrum: mCerulean3, mTurquoise2, mT-Sapphire, Clover, mKusabiraOrange2, mOrange2, LSS-mOrange, mRuby2, mBeRFP, mCardinal, iRFP670, NirFP, and iRFP720.

## 1. Introduction

In the past three decades, two-photon microscopy has become an irreplaceable method in biomedical research to study dynamic cellular processes in living organs and organisms. Research in neurobiology [1], cancer [2], immunity and autoimmunity [3,4,5,6], or neuroinflammatory and neurodegenerative diseases [7,8] especially benefit from it.

Lower energy wavelengths are used to reach deeper regions in intact tissue, e.g., approximately 500 µm in the brain cortex, a depth which is less susceptible to tissue scattering and causes less bleaching and phototoxicity [9]. In particular, multiplexing techniques [10,11,12,13] gain even deeper insights into the complex interplay of cells and structures within tissues by exciting them with multiple laser lines simultaneously, providing real-time access to more fluorophores to label structures and cell populations.

The structural and thus functional stability of fluorophores over a period of several weeks, besides the resolution and imaging depth, is an important requirement for intravital imaging to investigate the long-term development of diseases, such as by the longitudinal intravital imaging of the bone marrow (LIMB) system [14,15]. For long-term experiments, fluorescent proteins that can be expressed in specific cell populations in transgenic animals are more commonly used than synthetic fluorescent dyes. The concentration of fluorescent proteins in the cells remains constant even when the cells divide over time, causing less cellular stress or damage due to dye toxicity.

To benefit optimally from two-photon excitation, it is essential to know the two-photon excitation properties of fluorescent proteins. In the past, much focus was set on two-photon excitation spectroscopy, for example, how to determine the two-photon excitation cross sections [16], how to expand the wavelength range [17], or under which conditions they are stable [18]. In the course of this, many two-photon spectra of fluorescent dyes [19] and proteins [20,21] known at that time have been published. However, in the meantime, several new fluorescent proteins have been developed, especially in the near-infrared range, as well as some derivatives of blue, cyan, and green fluorescent proteins, whose two-photon spectra, to our knowledge, are not found in the literature [22] or in databases such as FPbase [23]. At most, the two-photon spectra of very bright synthetic dyes in the red/infrared fluorescence range, such as those needed for STED, have been published [24].

Due to different symmetry-based selection rules, i.e., different quantum mechanical backgrounds [25,26], the two-photon spectra often differ from their one-photon counterparts in shape and position [20]. It is proven that the peaks of two-photon spectra are always blue-shifted and never shifted to higher wavelengths (blue shift) [20]; thus, doubling the single-photon excitation wavelength is not sufficient, and the two-photon spectrum cannot be predicted properly from the single-photon spectrum [18]. Proteins have a three-dimensional folding structure, which determines the protein function [27]. The ion-, salt content, presence of other proteins, and pH-value [18,28], respectively, influence this protein structure and, thus, its function. Isolating the fluorescent proteins from their cellular environment may provoke errors in the protein folding and, thus, its function and properties, including photophysical properties.

Here we present normalized, dimensionless two-photon excitation spectra of various, not-yet-characterized fluorescent proteins in living cells to guarantee the right electrolytic environment. We used transfected HEK-293T-cells and isolated splenocytes from fluorescent reporter mice expressing the following fluorescent proteins: mCerulean3, mTurquoise2, mT-Sapphire, Clover, mKusabiraOrange2, mOrange2, LSS-mOrange, mRuby2, mBeRFP, mCardinal, iRFP670, NirFP, iRFP720. The spectra were measured in a wide wavelength range by means of a titanium-sapphire laser (Ti:Sa, 760 ≤ λ_Ti:Sa_ ≤ 1040 nm) and an optical parametric oscillator (OPO, 1060 nm ≤ λ_OPO_ ≤ 1300 nm). We used the known two-photon spectra of mNeonGreen [29], mAmetrine [29], and mKate2 [20] as references.

## 2. Results

The two-photon spectra of the fluorescent proteins were acquired over a broad excitation wavelength range, combining the Ti:Sa (760 ≤ λ_Ti:Sa_ ≤ 1040 nm) and OPO (1060 ≤ λ_OPO_ ≤ 1300 nm) tunning ranges in 10 nm steps. For each wavelength, three images were acquired at the same settings, so that the measurement uncertainty can be estimated subsequently. The laser power is selected with a continuous power attenuator (a λ/2 plate in front of the beam splitter) as well as in the respective beam path of the Ti:Sa laser and the OPO in such a way that saturation of the PMT is avoided, but the fluorophore is still efficiently excited.

To preserve continuous transition from the Ti:Sa to the OPO tunning range, the raw data GV_F_(λ) were corrected for background signal GV_BG_(λ) and normalized for squared peak photon flux density Φ^2^(λ). We defined F_2PE_(λ),the photon-flux-density-normalized two-photon excited fluorescence signal, as follows:

(1) $$F_{2\mathrm{PE}}\left(\lambda \right)=\frac{{\mathrm{GV}}_{F}\left(\lambda \right)-{\mathrm{GV}}_{\mathrm{BG}}\left(\lambda \right)}{\Phi^{2}\left(\lambda \right)}$$

The photon flux density Φ(λ) is the number of photons #_Ph_ per time unit t and per excitation area A:

(2) $$\Phi \left(\lambda \right)=\frac{{\#}_{\mathrm{Ph}}}{t\cdot A}$$

The radiant power P(λ) is the energy of a photon E(λ) multiplied by the number of photons #_Ph_ per time unit t:

(3) $$P\left(\lambda \right)=\frac{E_{\mathrm{Ph}}\cdot {\#}_{Ph}}{t}$$

From the Equations (2) and (3) results, the mean photon flux:

(4) $$\Phi_{\mathrm{mean}}\left(\lambda \right)=\frac{P\left(\lambda \right)}{A\cdot E_{\mathrm{Ph}}}$$

Since the excitation sources used here are pulsed lasers, the photon flux density in the peak is required. Multiplying the mean photon flux density (Equation (4)) by the repetition rate of the laser RR and the pulse width τ_p_ gives the photon flux density in the peak:

(5) $$\Phi_{\mathrm{Peak}}\left(\lambda \right)=\frac{P}{A\cdot E_{Ph}\cdot \mathrm{RR}\cdot \tau_{P}}$$

The diffraction limits of a two-photon microscope define excitation in the focus. We assume that the focus is circular, so the area is A = π∙r^2^. For the radius at the focus r, half the lateral resolution of the microscope d_x,y_ can be used.

(6) $$d_{x,y}=\frac{1.22\cdot \lambda }{2\cdot \sqrt{2}\cdot N.A.}\approx \frac{0.431\cdot \lambda }{N.A.}$$

and Equation (5) becomes:

(7) $$\Phi_{\mathrm{Peak}}\left(\lambda \right)=\frac{P\left(\lambda \right)}{\mathrm{RR}\cdot \tau_{P}\cdot \pi \cdot {\left(\frac{\lambda \cdot 0.431}{2\cdot N.A.}\right)}^{2}\cdot E_{Ph}\left(\lambda \right)}$$

Substituting Equation (7) into Equation (1) and using photon energy E_ph_(λ) = hc/λ, with h as the Planck constant and c the speed of light, yields the following corrected fluorescence signal:

(8) $$F_{2PE}\left(\lambda \right)=\frac{{\mathrm{GV}}_{F}\left(\lambda \right)-{\mathrm{GV}}_{\mathrm{BG}}\left(\lambda \right)}{P^{2}\left(\lambda \right)}\cdot h^{2}c^{2}\cdot {\mathrm{RR}}^{2}\cdot \tau_{P}^{2}\cdot \pi^{2}\cdot \frac{\lambda^{2}\cdot 0.431^{4}}{16\cdot N.A.^{4}}$$

For Equations (1)–(8): F_2PE_(λ) is the photon-flux-density-normalized two-photon excited fluorescence signal of the excitation wavelength λ (=excitation efficiency). GV_F_(λ) is the gray value after binary-masking for the fluorescence signal. GV_BG_(λ) is the gray value of the background acquired by the inverted fluorescence signal mask. P is the excitation power, monitored simultaneously during each measurement for each wavelength by reflecting a small amount of the excitation light (approx. 5%) into a power meter (see Section 4). This was correlated to the laser power under the objective lens measured by the power meter, previously. h is the Planck constant, c is the speed of light, RR is the repetition rate of the lasers, τ_P_ is the laser pulse width, and N.A. is the numerical aperture of the objective lens.

Equation (8) is very similar to Equation (13) in reference [17], but it considers the circular excitation area defined by the diffraction limit and takes the repetition rate RR into account. In our case, the Ti:Sa laser (80 MHz) pumps the OPO (thus also 80 MHz), and the RR is not wavelength-dependent. However, when the excitation wavelength range includes laser sources with different repetition rates, the RR in the equation becomes relevant. The pixel dwell time ($t_{dwell}={\#}_{pulses}\cdot \tau_{p}$), was not considered here, since all raw data were acquired with the same recording settings which also includes things such as the detector gain or pixel size. Furthermore, we assumed that the fluorophore concentration is constant and not decreased by saturation or photobleaching.

The corrected signal F_2PE_(λ) was normalized with the maximum of the respective spectrum to a dimensionless curve.

**Figure 1:**
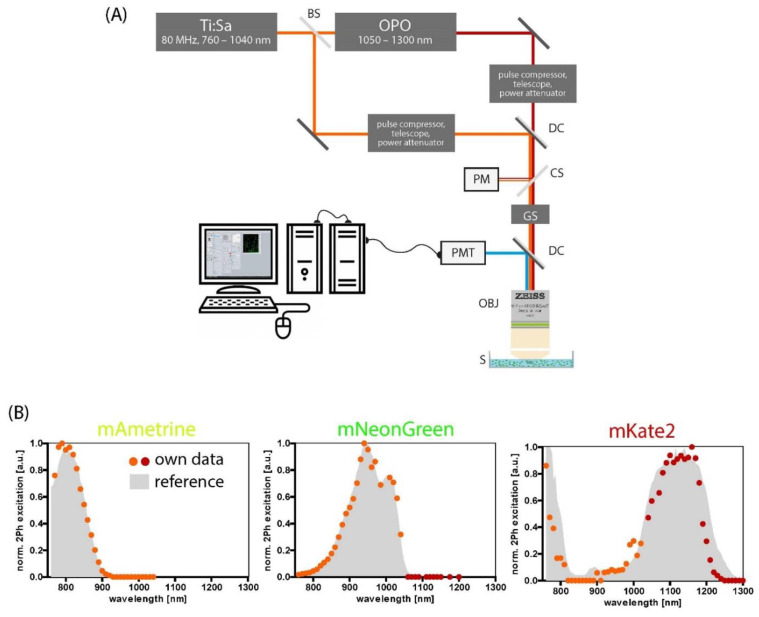
Acquisition and evaluation of two-photon excitation spectra of fluorescent proteins expressed in the cytosol of transfected HEK-293T cells. (**A**) Experimental setup. The beam of a titanium sapphire laser (Ti:Sa) at 760–1040 nm passes a beam splitter (BS), one part of the laser beam pumps an optical parametric oscillator (OPO) to generate higher wavelengths (1060–1300 nm). Both Ti:Sa and OPO beams each travel through a beam shaper including a pulse compressor, a telescope, and a lambda half-plate for power attenuation and are combined by a dichroic mirror (DC). To monitor the excitation power, a cover slip (CS) is placed at 45° to the optical axis and reflects approx. 5% of the laser light to the probe of a power meter (PM). The galvo scanner (GS) scans the remaining 95% of the beam over the sample. The laser beams are focused by a water immersion objective lens (OBJ). Another dichroic mirror separates excitation and emission light. The emission light is detected by a photomultiplier tube (PMT). The signal from the PMT is transferred into a computer, where it is digitized and reconstructed into an image. (**B**) mAmetrine, mNeonGreen, and mKate2 serve as proof of principle. Dots represent our own data recorded in the Ti:Sa (orange) and OPO (dark red) wavelength range, and the gray-filled curves represent reference data adapted from FPbase [23,30] The color of the designations illustrates the emission wavelength (see Table 2 in the Section 4).

Figure 1A shows the experimental setup, described in the material and methods section. Figure 1B presents the two-photon spectrum of mAmetrine, mNeonGreen, and mKate2 in the wide Ti:Sa and OPO tunning range. They serve as references for our measurement setup, data acquisition, and analysis and agree well within the measurement uncertainty with the spectrum found in the literature: mAmetrine and mNeonGreen [29], mKate2 [20], and data adapted from FPbase [23,30]. The measurement uncertainty for the wavelengths is Δλ_Ti:Sa_ = ±5 nm in the Ti:Sa range and Δλ_OPO_ = ±12 nm in the OPO range, which is a bad compromise of ±9 nm Δλ_OPO_ ±13 nm. The uncertainty of the excitation efficiency is ΔF_2PE_(λ) = ±0.075 a.u., obtained by the propagation of uncertainty (Gaussian error), including the measurement uncertainty of the involved experimental setup as provided by the manufacturer.

Before acquiring the spectra of not-yet-characterized fluorescent proteins, we ensured through the example of several proteins that the observed fluorescence is induced by a pure two-photon excitation. Therefore, we measured the dependence of the fluorescence signal on the laser power, to prove it is quadratic using the logarithmic laws for powers $\log_{b}\left(x^{p}\right)={p\;\log}_{b}\;x$ (x is the number to be taken the logarithm, b is the basis, p the logarithmic power).

**Figure 2:**
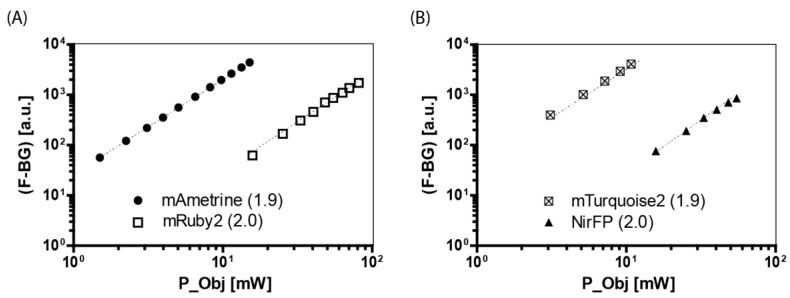
Logarithmic dependence of the background-corrected fluorescence (F-BG) generated by two-photon excitation on the time-averaged excitation power entering the objective lens. (**A**) mAmetrine (black dots) and mRuby2 (unfilled squares). (**B**) mTurquoise (crossed squares) and NirFP (black triangles). The fluorescent proteins were expressed in the cytosol of transfected HEK-293T cells. P_Obj is the excitation power measured under the objective lens. The dotted lines are the linear fits, and the slopes are given in parentheses.

The double-logarithmic representations in Figure 2 can be approximated by linear functions with a slope between 1.9 to 2.1, indicating two-photon excitation processes (Table 1).

**Figure 3:**
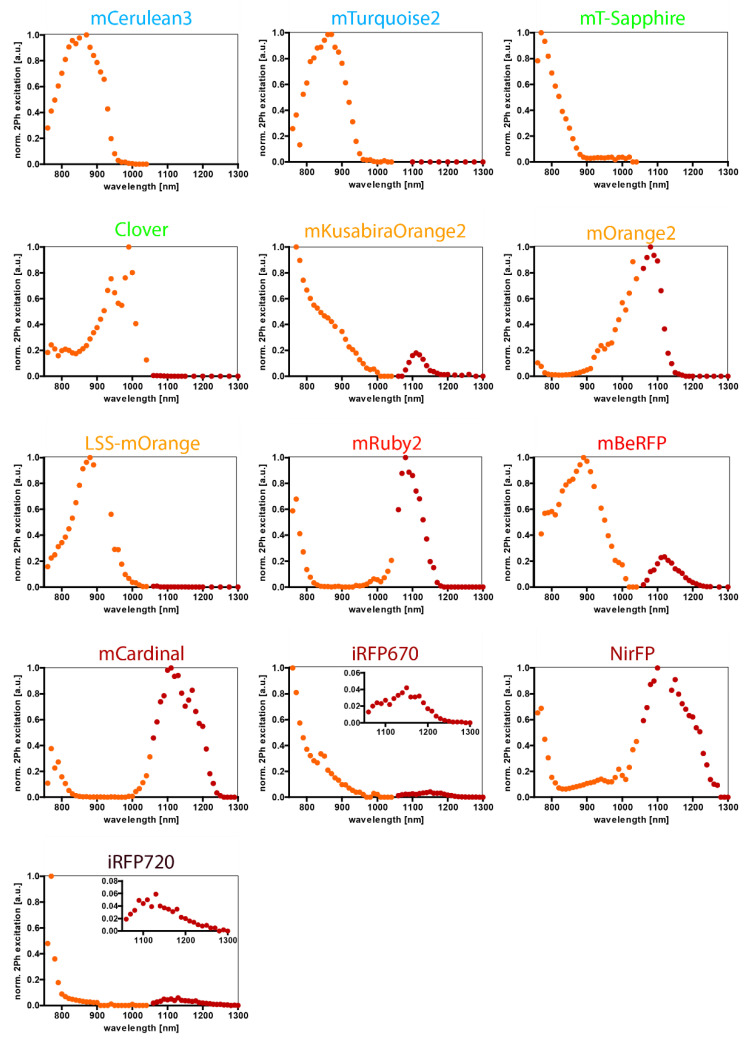
Two-photon excitation spectra of various fluorescent proteins expressed in the cytosol of transfected HEK-293T cells and, in the case of mKusabiraOrange, in isolated splenocytes from fluorescent reporter mice. Normalized excitation efficiency in arbitrary units (a.u.) are plotted versus the excitation wavelength in nanometers (nm). The fluorophores were excited by the Ti:Sa laser (760–1040 nm; orange dots) and OPO pumped by Ti:Sa (1060–1300 nm; dark-red dots). The spectra are ordered according to their emission maximum from the blue to deep red/infrared spectral range, illustrated by the color of the designations. A table of the spectra in numbers can be found in the Supplementary Material Tables S1 and S2.

Figure 3 shows the resulting normalized two-photon excitation spectra, corrected for the photon flux density and the background of the fluorescent proteins mCerulean3, mTurquoise2, mT-Sapphire, Clover, mKusabiraOrange2, mOrange2, LSS-mOrange, mRuby2, mBeRFP, mCardinal, iRFP670, NirFP, iRFP720. To express the fluorescent proteins, we used transfected HEK cells. This was the case for almost all proteins considered, except for mKusabiraOrange2. There, we used splenocytes from transgenic mice B1-8^+/+^ Jκ^−/−^ Kusabira Orange mice as a reliable source of cells, already expressing the needed fluorophore.

## 3. Discussion

In intravital two-photon imaging experiments, knowledge on two-photon excitation spectra of fluorescent samples is needed to select the optimal laser line, to effectively excite as many fluorophores as possible, in the considered sample. Increasing the number of excitable fluorophores means enlarging the number of cell populations and tissue compartments to be studied and better accessing their location and interplay in complex systems such as primary and secondary lymphoid organs.

Here, we presented normalized two-photon spectra of various newer, not-yet-characterized red and near-infrared fluorescent proteins as well as derivatives of known cyan, blue, and green fluorescent proteins in a broad spectral range covering both Ti:Sa and OPO wavelengths. For comparison, we showed the spectra we could find on pertinent databases, such as FPbase [23,30], along with our data. Compared to these data our spectra recorded in the Ti:Sa range, namely, mNeonGreen and mAmetrine, they are of high accuracy. The spectrum of mKate2 reaching over the Ti:Sa and OPO range is within the error margin of good agreement with the comparison data.

We found an incomplete two-photon excitation spectrum of mCardinal in the supplementary of Chu et al., (2014) [31] measured on purified proteins in the Ti:Sa wavelength range. These data are consistent with ours in this range, but the expansion of the excitation range to OPO wavelengths of our spectra reveals the maximum peak of mCardinal at higher wavelengths. Adhikari et al., (2021) [32], among others, present normalized two-photon excitation spectra of fluorescent proteins of some of the proteins we also measured, namely, mCerulean3 and mTurquoise. These spectra were measured in a PAA gel doped with fluorescent proteins at pH 8, not in a cellular environment as in the present work. Physiological intracellular pH lays usually between 7.0 and 7.4 [33]. Despite the different experimental conditions, i.e., extracellular vs. intracellular and pH 8 vs. intracellular pH, the spectra of mCerulean3 and mTurquoise measured by Adhikari et al., (2021) and our spectra are similar, yet not identical. Within the measurement uncertainty, the spectra peak at the same wavelengths and cover the same spectral range, showing differences only in their shape. As it was shown that pH is critical for the excitation and emission spectra of fluorescent proteins [18], because the folding structure of a protein may be altered by pH, this may explain the differences between the spectra published by Adhikari et al., (2021) and our spectra.

The advantage of recording the two-photon spectra over a long wavelength range and studying the fluorescent protein in its cellular environment is that all relevant excitation shoulders are found and the measurement conditions are similar to those in intravital experiments.

The spectra were measured indirectly by estimating the two-photon excitation efficiency by increasing or decreasing emission light at different excitation wavelengths. Since the spectra were measured in intact living cells, autofluorescent molecule species in the cell could have influenced the resulting spectra. For example, the maximum of the emission light of the ubiquitous metabolic co-enzymes NADH and NADPH at 467 nm (100%) reaches from 440 nm to 516 nm (70%) and may thus interfere with the emission light of mAmetrine or other blue and cyan proteins. The probability of a two-photon absorption process of a fluorescent molecule is given by the two-photon action cross-section σ_2PE_η_2PE_, the product of the fluorescence quantum yield η_2PE_ and the absolute two-photon absorption cross-section σ_2PE_ [9]. In contrast to fluorescent proteins expressed by transfected cells, the two-photon action cross-section of the intrinsic co-enzymes NAD(P)H is with σ_2PE_ η_2PE_ (760 nm) ≤ 0.01 GM [34] (Göppert-Mayer units: 1 GM = 10^−50^ cm^4^ s photon^−1^ molecule^−1^), comparatively low in contrast to those such as mAmetrine with σ_2PE_ η_2PE_ (760 nm) ≈ 37 GM [20]. The same applies to flavins, such as FAD, in the green emission range. Thus, the influence of autofluorescent molecules can be neglected.

In order to assess the spectra of the proteins in absolute two-photon action cross-section values calculated using Equation 15 in Xu et al., 1996, the fluorophore concentration expressed in cells is needed. The fluorophore concentration can be determined, for example, by fluorescence correlation spectroscopy (FCS) [35,36,37,38]. This method measures the concentration of fluorescent molecules, which fluctuate in and out of the two-photon excitation volume. FCS is a very elegant solution since it measures the concentration based on the property considered for the spectra—their fluorescence—and thus excludes all non-functionally folded (thus, non-fluorescent) protein molecules. This approach was already used to measure the fluorophore concentration in order to calculate the two-photon action cross-section [9,39]. Despite its elegancy, FCS is very susceptible to a high fluorophore concentration or entrapped fluorophores, as both influence the autocorrelation curve G(τ). We aim to establish FCS in our cells to determine the absolute concentration of FPs, accounting for their subcellular location, e.g., freely diffusing in the cytosol or being bound to actin filaments. However, in the present work, we have kept our focus to relative two-photon spectra.

The two-photon spectra of the infrared fluorescent proteins iRFP670 and iRFP720 are remarkably similar, which can be explained by their common origin. Both are derivatives of the natural fluorescent protein in the bacteria *Rhodopseudomonas palustris* [40]. Like iRFP670, the fluorescence maximum of NirFP is at 670 nm. However, NirFP is a derivative of Katushka [41], whose origin is the natural fluorescent protein in the anemone *Entacmaea quadricolor* (*EQ*), and thus, is not at all related to iRFP670, which is also evident in the two-photon spectrum. NirFP is rather distantly related to mKate, whose derivatives include mCardinal [31], mBeRFP—a long-stokes-shift variant—[42] and mKate2 [41]. Similar to Katushka, mKate also originated from TurboRFP, a derivative of the natural fluorescent protein in the anemone *EQ. EQ* is also the origin of mRuby, but here, the base is eqFP611 rather than eqFP578, as mentioned for the other red fluorescent proteins. The orange fluorescent proteins mOrange2 and the long-stokes-shift variant LSS-mOrange are descendants of DsRed, the natural fluorescent protein of the anemone *Discosoma*. In contrast, the orange fluorescent protein mKusabira-Orange2 is derived from fluorescent protein in the coral *Fungia concinna* [43] and is thus unrelated to the other orange fluorescent proteins also seen in the dissimilar two-photon spectra. Most of the green and cyan fluorescent proteins that are presented in this work, namely, mAmetrine [44], mCerulean3 [45], mTurqouise2 [46], mT-Sapphire [44], and Clover [47], are derivatives of avGFP, the green fluorescent protein in the jellyfish *Aequorea victoria*. The green fluorescent protein mNeonGreen [48] is not related to them, and originates from the natural fluorescent protein LanYFP in the lancelet fish *Branchiostoma lanceolatum.* Interestingly the two-photon spectrum of mNeonGreen, with its two maxima, is quite similar to the one of Clover, although the two are not related, as just explained. Concluding, the photophysical properties, here, two-photon excitation spectra, strongly correlate with the provenience of the FPs. If related, the spectra are similar or behave similarly, and if not, even for similar emission spectra, they differ, presumably, due to a different molecular structure of the chromophore, which leads to different selection rules governing the two-photon excitation process.

Multiplexing has recently become an important tool in microscopy, across various disciplines in life science. While it is being increasingly used in static histology, where dozens of markers can be analyzed in the same tissue [49,50], its application for deep tissue and intravital two-photon imaging is still limited to less than 10 markers [10]. The data presented here provide a valuable resource for expanding the possible combinations of fluorescent proteins in two-photon microscopy.

## 4. Material and Methods

### 4.1. Two-Photon Laser-Scanning Microscope Setup

Two-photon fluorescence imaging experiments were performed as previously described [51], using a specialized laser-scanning microscope based on a commercial scan head (TriMScope II, LaVision BioTec, Bielefeld, Germany). The experimental setup is depicted in Figure 1A. A near-infrared, mode-locked titanium sapphire laser (Ti:Sa, Chameleon Ultra II, Coherent, Dieburg, Germany) and an infrared optical parametric oscillator (OPO, APE, Berlin, Germany) were used as excitation sources. The repetition rate of both sources was 80 MHz, and the pulse width under the objective lens was 247 fs for Ti:Sa at 850 nm and 166 fs for OPO at 1107 nm (pulseCheck, APE, Berlin, Germany). The Ti:Sa and OPO beams, both linearly polarized, were combined in the scan head using a dichroic mirror (T1045, Chroma, Bellows Falls, VT, USA). A water-immersion objective lens (20×, NA 1.05, Plan-Apochromat, Carl Zeiss, Jena, Germany) was used to focus both laser beams into the sample. The laser power was controlled by combinations of λ/2 wave plates and beam splitters. The emission signal was detected in the backward direction using a dichroic mirror (775 nm, Chroma, Bellows Falls, VT, USA) by a photomultiplier tube (H7422, Hamamatsu, Japan). We considered photobleaching of the fluorescent proteins by measuring the spectra in ascending and additionally in descending order of the wavelengths. Furthermore, we chose the excitation power such that the detectors were not saturated and phototoxicity and photobleaching were avoided, meaning an average maximum laser power of less than 10 mW in all imaging experiments. The time-averaged excitation power entering the objective lens was determined from the values of 5% laser beam reflection measured by the power meter (Newport Economical Handheld Laser Power Meter, 843-R, Israel) equipped with the sensor (Newport 818-ST2-IR Ge Metal Wand Detector, 780–1800 nm).

### 4.2. Samples

We transfected HEK-293T cells cultures following the protocol provided for Lipofectamine 3000 (ThermoFischer Scientific, Waltham, MA), using vectors encoding the fluorescent proteins listed in Table 2 (except mKusabira-Orange2). Each HEK-cell culture expressed a single fluorescent protein.

The spectrum of mKusabira-Orange2 was acquired in a culture of isolated splenocytes from B1-8^+/+^ Jκ^−/−^ Kusabira Orange mice (Tg(CAG-mKO2/CDT1)596Amiy crossed with C57BL/6-Prdm1tm1Nutt/J [52]). For the cell’s isolation, the spleen was cut into small pieces, pressed through a strainer and suspended in RPMI medium containing 10% FCS. Erythrocyte lysis buffer was added to the cell suspension to remove erythrocytes. The suspension was centrifuged and the pellet was resuspended in PBS [10].

## Figures and Tables

**Table 1:** Resulting slope per fluorescent protein expressed in the cytosol of transfected HEK-293T cells and excitation wavelength.

	λ [nm]	Slope
mTurquoise 2	800	1.9 ± 0.1
mAmetrine	800	1.9 ± 0.1
mRuby2	1100	2.0 ± 0.1
NirFP	1100	2.0 ± 0.1

**Table 2:** Plasmid manufacturer/transgenic mouse of the investigated fluorescent proteins with maximum of emission light.

Fluorescent Protein	Manufacturer Designation (Plasmid)	Item Number and Manufacturer	Emission Max [nm]
mCerulean3	mCerulean3-N1	54730, Addgene, Cambridge, MA	474
mTurquoise2	pmTurquoise2-N1	60561, Addgene, Cambridge, MA	474
mT-Sapphire	mT-Sapphire-N1	54569, Addgene, Cambridge, MA	511
Clover	pcDNA3-Clover	40259, Addgene, Cambridge, MA	516
mNeonGreen	pcDNA3.1-mNeonGreen-LEHD-NanoLuc	98289, Addgene, Cambridge, MA	517
mAmetrine	mAmetrine-N1	54505, Addgene, Cambridge, MA	526
mKusabira-Orange2	Speenocytes from B1-8^+/+^ Jκ^−/−^ Kusabira Orange mouse		561
mOrange2	*Kindly provided by Andreas Acs and Thomas H. Winkler, University of Erlangen-Nürnberg, Erlangen, Germany*		565
LSS-mOrange	pLSSmOrange-C1	37131, Addgene, Cambridge, MA	573
mRuby2	pcDNA3-mRuby2	40260, Addgene, Cambridge, MA	594
mBeRFP	pcDNA3.1-mBeRFP	175173, Addgene, Cambridge, MA	623
mKate2	*Kindly provided by Andreas Acs and Thomas H. Winkler, University of Erlangen-Nürnberg, Erlangen, Germany*		633
mCardinal	pcDNA3-mCardinal	51311, Addgene, Cambridge, MA	658
iRFP670	piRFP670-N1	45457, Addgene, Cambridge, MA	670
NirFP	pNirFP-c	FP741, Evrogen Joint Stock Company, Moscow, Russia	670
iRFP720	piRFP720-N1	45461, Addgene, Cambridge, MA	720

## Data Availability

Data are available upon request from the corresponding author.

## Supplementary Materials

The following supporting information can be downloaded at: https://www.mdpi.com/article/10.3390/ijms232113407/s1.
