# Supplementary material for: Two-Photon Excitation Spectra of Various Fluorescent Proteins within a Broad Excitation Range
Source: Int J Mol Sci. 2022 Nov 2;23(21):13407. doi: 10.3390/ijms232113407 (PMC9656010; doi:10.3390/ijms232113407)
Supplement: Supplementary file 1 [file ijms-23-13407-s001.zip › ijms-1960984-supplementary.pdf]

Supplement Table S1. Two-photon spectra in numbers. mCerulean3, mTurquoise2, mT-Sapphire, Clover, mNeonGreen, mAmetrine, mKusabiraOrange2, mOrange2

**wavelength λ_{ex} [nm]**	**mCerulean3**	**mTurquoise2**	**mT-Sapphire**	**Clover**	**mNeonGreen**	**mAmetrine**	**mKusabiraOrange2**	**mOrange2**
**760**	0,280	0,258	0,783	0,184	0,018			0,104
**770**	0,411	0,364	1,000	0,243	0,023	0,760	1,000	0,076
**780**	0,496	0,133	0,934	0,211	0,028	0,973	0,896	0,027
**790**	0,605	0,524	0,819	0,159	0,033	1,000	0,743	0,016
**800**	0,704	0,611	0,689	0,198	0,044	0,952	0,667	0,012
**810**	0,810	0,777	0,587	0,210	0,057	0,971	0,602	0,011
**820**	0,907	0,805	0,508	0,200	0,085	0,917	0,550	0,010
**830**	0,957	0,882	0,391	0,181	0,108	0,811	0,527	0,010
**840**	0,933	0,889	0,334	0,175	0,133	0,659	0,493	0,011
**850**	0,977	0,942	0,262	0,192	0,175	0,542	0,467	0,013
**860**	1,003	0,986	0,180	0,213	0,223	0,427	0,451	0,018
**870**	1,000	0,987	0,108	0,237	0,299	0,315	0,423	0,024
**880**	0,905	0,888	0,059	0,289	0,391	0,202	0,386	0,030
**890**	0,841	0,850	0,037	0,337	0,475	0,111		0,039
**900**	0,787	0,764	0,030	0,376	0,521	0,046	0,346	0,049
**910**	0,714	0,613	0,029	0,441	0,585	0,021	0,286	0,060
**920**	0,657	0,462	0,031	0,507	0,703	0,011	0,227	0,146
**930**	0,428	0,311	0,034	0,664	0,881	0,000	0,206	0,197
**940**	0,198	0,160	0,033	0,755	1,000	0,000	0,179	0,237
**950**	0,081	0,065	0,031	0,646	0,954	0,000	0,128	0,211
**960**	0,030	0,020	0,035	0,565	0,822	0,000	0,098	0,247
**970**	0,016	0,015	0,037	0,549	0,863	0,000	0,063	0,258
**980**	0,016	0,017	0,018	0,761	0,690	0,000	0,051	0,360
**990**	0,007	0,005	0,035	1,000		0,000	0,056	0,437
**1000**	0,005	0,000	0,037	0,802		0,000	0,031	0,569
**1010**	0,000	0,000	0,027	0,407	0,745	0,000	0,000	0,513
**1020**	0,000	0,010	0,038		0,709	0,000	0,000	0,643
**1030**	0,000	0,000	0,000		0,590	0,000	0,000	0,886
**1040**	0,000	0,000	0,000	0,126	0,319	0,000	0,000	0,755
**1060**				0,006	0,000		0,000	0,835
**1070**				0,005	0,000		0,000	0,919
**1080**				0,004	0,000		0,049	1,000
**1090**				0,002	0,000		0,107	0,935
**1100**		0,000		0,001			0,160	0,892
**1110**				0,000	0,000		0,180	0,662
**1120**				0,000	0,000		0,165	0,366
**1125**		0,000						
**1130**				0,000	0,000		0,131	0,178
**1140**				0,000	0,000		0,082	0,098
**1150**		0,000		0,000	0,000		0,044	0,029
**1160**							0,036	0,018
**1170**							0,024	0,009

**1175**		0,000		0,000	0,000			
**1180**							0,014	0,000
**1190**							0,012	0,000
**1200**		0,000		0,000	0,000		0,014	0,000
**1220**							0,010	0,000
**1225**		0,000		0,000				
**1240**							0,007	0,000
**1250**		0,000		0,000				
**1260**							0,015	0,000
**1275**		0,000		0,000				
**1280**							0,000	0,000
**1300**		0,000		0,000			0,000	0,000

Supplement Table S2. Two-photon spectra in numbers. LSS-mOrange, mRuby2, mBeRFP, mKate2, mCardinal, iRFP670, NirFP, iRFP720

**wave-length λ_{ex} [nm]**	**LSS-mOrange**	**mRuby2**	**mBeRFP**	**mKate2**	**mCardinal**	**iRFP670**	**NirFP**	**iRFP720**
**760**	0,152	0,588		0,861	0,109	1,000	0,653	0,480
**770**	0,217	0,679	0,410	0,474	0,376	0,810	0,688	1,000
**780**	0,240	0,411	0,569	0,391	0,226	0,575	0,449	0,360
**790**	0,299	0,271	0,574	0,168	0,273	0,460	0,305	0,177
**800**	0,329	0,135	0,583	0,169	0,158	0,371	0,154	0,089
**810**	0,370	0,076	0,557	0,121	0,106	0,322	0,111	0,069
**820**	0,432	0,033	0,637	0,000	0,052	0,283	0,072	0,054
**830**	0,510	0,016	0,742	0,000	0,026	0,267	0,065	0,048
**840**	0,626	0,006	0,789	0,000	0,013	0,336	0,064	0,042
**850**	0,754	0,004	0,817	0,000	0,006	0,319	0,069	0,037
**860**	0,879	0,004	0,833	0,000	0,004	0,210	0,075	0,032
**870**	0,927	0,001	0,894	0,000	0,005	0,183	0,082	0,028
**880**	0,961	0,004	0,945	0,000	0,001	0,150	0,089	0,027
**890**	0,908	0,006	1,000	0,000	0,002	0,132	0,096	0,023
**900**		0,000	0,972	0,058	0,002	0,098	0,107	0,023
**910**		0,000	0,891	0,000	0,002	0,096	0,112	0,000
**920**		0,000	0,776	0,061	0,001	0,070	0,119	0,000
**930**		0,000		0,067	0,000	0,053	0,131	0,000
**940**	0,539	0,012	0,609	0,082	0,003	0,039	0,140	0,011
**950**	0,279	0,009	0,516	0,071	0,002	0,039	0,129	0,000
**960**	0,277	0,013	0,395	0,078	0,000	0,022	0,119	0,000
**970**	0,170	0,022	0,314	0,082	0,000	0,000	0,120	0,000
**980**	0,093	0,042	0,206	0,126	0,000	0,000	0,153	0,000
**990**	0,065	0,063	0,190	0,269	0,007	0,030	0,217	0,000

**1000**	0,036	0,057	0,172	0,297	0,007	0,023	0,169	0,009
**1010**	0,031	0,041	0,065	0,189	0,044	0,000	0,138	0,000
**1020**	0,016	0,072	0,000	0,278	0,066	0,000	0,231	0,000
**1030**	0,004	0,122	0,000		0,113	0,000	0,368	0,000
**1040**	0,004	0,206	0,000	0,472	0,166	0,000	0,431	0,000
**1050**				0,596	0,313			
**1060**	0,005	0,597	0,018		0,459	0,013	0,593	0,019
**1070**	0,006	0,878	0,053	0,657	0,583	0,020	0,694	0,027
**1080**	0,001	1,000	0,120	0,808	0,739	0,024	0,873	0,033
**1090**	0,001	0,887	0,132	0,882	0,785	0,023	0,900	0,049
**1100**	0,001	0,861	0,180	0,938	0,983	0,027	1,000	0,044
**1110**	0,001	0,741	0,228	0,885	1,000	0,022	1,027	0,050
**1120**	0,001	0,681	0,234	0,910	0,935	0,029	1,046	0,039
**1130**	0,001	0,519	0,206	0,937	0,942	0,033	1,036	0,059
**1140**	0,001	0,370	0,185	0,910	0,805	0,036	0,828	0,040
**1150**	0,001	0,197	0,141	0,922	0,705	0,042	0,910	0,037
**1160**	0,000	0,150	0,125	1,000	0,752	0,031	0,798	0,035
**1170**	0,000	0,035	0,106	0,917	0,828	0,031	0,724	0,031
**1180**	0,000	0,009	0,072	0,732	0,664	0,032	0,681	0,035
**1190**	0,000	0,000	0,051	0,425	0,571	0,024	0,632	0,022
**1200**	0,000	0,000	0,035	0,294	0,549	0,017	0,621	0,020
**1210**		0,000	0,025	0,155	0,374	0,014	0,538	0,016
**1220**		0,000	0,014	0,063	0,182	0,008	0,507	0,014
**1225**	0,000							
**1230**		0,000	0,007	0,038	0,107	0,005	0,339	0,010
**1240**		0,000	0,003	0,015	0,034	0,003	0,250	0,008
**1250**	0,000	0,000	0,003	0,000	0,012	0,002	0,137	0,009
**1260**		0,000		0,000	0,000	0,001	0,101	0,005
**1270**		0,000		0,000	0,000	0,001	0,093	0,005
**1275**	0,000		0,000					
**1280**		0,000		0,000	0,000	0,001	0,000	0,000
**1290**		0,000		0,000	0,000	0,000	0,000	0,002
**1300**	0,000	0,000	0,000	0,000		0,000	0,000	0,000
